# Local DNA methylation helps to regulate muscle sirtuin 1 gene expression across seasons and advancing age in gilthead sea bream (*Sparus aurata*)

**DOI:** 10.1186/s12983-020-00361-1

**Published:** 2020-05-15

**Authors:** Paula Simó-Mirabet, Erick Perera, Josep Alvar Calduch-Giner, Jaume Pérez-Sánchez

**Affiliations:** Nutrigenomics and Fish Growth Endocrinology Group, Institute of Aquaculture Torre de la Sal, IATS-CSIC, 12595 Ribera de Cabanes s/n, Castellón, Spain

**Keywords:** Fish, Sirtuins, DNA methylation, Epigenetic marks, Skeletal muscle, Age, Season

## Abstract

**Background:**

Sirtuins (SIRTs) are master regulators of metabolism, and their expression patterns in gilthead sea bream (GSB) reveal different tissue metabolic capabilities and changes in energy status. Since little is known about their transcriptional regulation, the aim of this work was to study for the first time in fish the effect of age and season on *sirt* gene expression, correlating expression patterns with local changes in DNA methylation in liver and white skeletal muscle (WSM).

**Methods:**

Gene organization of the seven *sirts* was analyzed by BLAT searches in the IATS-CSIC genomic database (www.nutrigroup-iats.org/seabreamdb/). The presence of CpG islands (CGIs) was mapped by means of MethPrimer software. DNA methylation analyses were performed by bisulfite pyrosequencing. A PCR array was designed for the simultaneous gene expression profiling of *sirts* and related markers (*cs*, *cpt1a*, *pgc1α*, *ucp1,* and *ucp3*) in the liver and WSM of one- and three-year-old fish during winter and summer.

**Results:**

The occurrence of CGIs was evidenced in the *sirt1* and *sirt3* promoters. This latter CGI remained hypomethylated regardless of tissue, age and season. Conversely, DNA methylation of *sirt1* at certain CpG positions within the promoter varied with age and season in the WSM. Among them, changes at several SP1 binding sites were negatively correlated with the decrease in *sirt1* expression in summer and in younger fish. Changes in *sirt1* regulation match well with variations in feed intake and energy metabolism, as judged by the concurrent changes in the analyzed markers. This was supported by discriminant analyses, which identified *sirt1* as a highly responsive element to age- and season-mediated changes in energy metabolism in WSM.

**Conclusions:**

The gene organization of *SIRTs* is highly conserved in vertebrates. GSB *sirt* family members have CGI- and non-CGI promoters, and the presence of CGIs at the *sirt1* promoter agrees with its ubiquitous expression. Gene expression analyses support that *sirts*, especially *sirt1*, are reliable markers of age- and season-dependent changes in energy metabolism. Correlation analyses suggest the involvement of DNA methylation in the regulation of *sirt1* expression, but the low methylation levels suggest the contribution of other putative mechanisms in the transcriptional regulation of *sirt1*.

## Background

Aquaculture is a fast-growing food production sector [1], but the maintenance of the current growing trend will rely on a deeper understanding of the genetic and downstream physiological mechanisms affecting productive traits. Biomarkers that identify and follow up desired traits are especially appropriate for the selection of environmental conditions and genotypes that promote or exhibit better physiological performances [2–4]. This is of particular relevance for productive traits related to intermediary metabolism that are not easy to measure (e.g., feed efficiency, energy status, redox homeostasis). Gene expression patterns of growth-promoting factors and antioxidant markers or lipid- and energy-metabolism-related markers have become highly informative for disclosing different metabolic features in challenged fish and higher vertebrates [5–7]. In this sense, sirtuins (SIRTs) are a conserved family of enzymes that couple protein deacylation with the energy status of the cell via the cellular NAD^+^/NADH ratio, linking nutrition and energy status with epigenetic regulation [8–10]. Particularly in gilthead sea bream (*Sparus aurata*) (GSB), the patterns of *sirt* gene expression are powerful biomarkers at the tissue-specific level, as they disclose different energy statuses resulting from nutrient availability or growth potentiality [4, 11, 12].

On the other hand, there is a growing interest in epigenetic markers because they are relatively stable and provide information about gene function and environmental interactions [13, 14]. Epigenetic mechanisms include changes in DNA methylation, histone modifications and noncoding RNA regulation that collectively affect chromatin architecture and the accessibility of the transcriptional machinery to genetic loci [15, 16]. Concretely, DNA methylation at promoter regions reduces gene expression by impairing the binding of transcriptional activators, whereas histone acetylation generally provides a permissive environment for transcription [17, 18], also as part of the DNA demethylation machinery [19]. Differentiated cells develop a relatively stable and unique DNA methylation pattern that regulates tissue-specific gene transcription and the precise tuning of different biological processes, particularly under conditions where the environment is manipulated or natural variation exists through life cycle or livestock production [20]. In fish, good examples of this are sex determination in European sea bass [21] and tongue sole [22], early maturation [23] and muscle development in Atlantic salmon [24], larval metamorphosis in the sea lamprey [25], growth traits and osmotic regulation in the tongue sole [26, 27], migration propensity in rainbow trout [28], and adaptive plasticity to freshwater and marine conditions in stickleback [29]. In GSB, there is abundant literature showing the effects of temperature on different aspects of intermediary metabolism, including changes in the expression of TFs, membrane translocases, molecular chaperones, and rate-limiting enzymes of fatty acid β-oxidation and the tricarboxylic acid cycle [30, 31]. However, how temperature and other biotic and abiotic factors affect the local DNA methylation of *sirts* or other key regulatory genes of energy metabolism remain poorly studied in fish. In contrast, there is abundant literature on the aging-mediated effects of DNA methylation and SIRT regulation and function in humans and rodents [32].

Since methylated cytosines are found primarily at CpG dinucleotides and CpG-rich regions (called CG islands, CGIs) span a number of promoters of annotated genes in higher vertebrates [33], the double aim of the present study was i) to map CGIs across the gene sequences of GSB *sirts* and ii) to correlate changes in gene expression and CGI methylation signatures using one- and three-year-old fish sampled in winter and summer as experimental models. Liver and white skeletal muscle (WSM) were chosen as target tissues because of their central role in fish metabolism, to provide new insights into the regulation and action of *sirts* in protandric hermaphroditic GSB, which is now highly cultured throughout the Mediterranean region.

## Methods

### Experimental fish, husbandry conditions and sampling

One- (+ 1) and three- (+ 3) year-old GSB of Atlantic origin (‘strain 1’ in [4]) were reared at the indoor experimental facilities of the Institute of Aquaculture Torre de la Sal (IATS-CSIC) in 3000-L tanks under natural photoperiod and temperature conditions at the IATS-CSIC latitude (40°5 N; 0°10E). Water temperature ranged from 10 °C in winter to 27 °C in summer. The water oxygen concentration was always higher than 75% saturation, and unionized ammonia remained below toxic levels (< 0.02 mg/L) irrespective of season. Fish were fed a standard commercial diet (EFICO YM 568; BioMar, Dueñas, Spain) once a day until visual satiety (3, 5 or 6 times per week depending on season and fish size). At the winter and summer sampling points, 10 fish per age class (class + 1, 50–115 g body weight; class + 3, 1 kg body weight) were anesthetized with 3-aminobenzoic acid ethyl ester (MS-222, 100 μg/mL), and the liver and WSM were rapidly excised, frozen in liquid nitrogen and stored at − 80 °C until RNA and DNA extraction.

### In silico analyses

Gene organization of GSB *sirts* was analyzed by BLAT searches in the IATS-CSIC genomic database of GSB (http://nutrigroup-iats.org/seabreamdb/). The retrieved sequences were manually curated by aligning genome sequences (Clustal X) with GSB *sirts* transcripts [11], using the online tool FGENESH from softberry for predicting gene structure [34]. For comparative purposes, the seven human and zebrafish SIRT counterparts were obtained from the ENSEMBL database (www.ensembl.org). Graphical representations were carried out with the online tool Exon-Intron Graphic Maker (http://wormweb.org/exonintron). Polyadenylation sites were identified by means of the Softberry POLYAH (www.softberry.com). Predictions of putative transcription start sites (TSSs) were performed by means of Promoter 2.0 (www.cbs.dtu.dk/services/Promoter/) [35]. Core promoter regions were predicted by using two complementary approaches: the Easy Promoter Prediction Program (EP3), which uses the GC content and structural features of DNA to identify promoter regions [36], and MatInspector (www.genomatrix.de), which searches transcription factor binding sites (TFBSs) and TFBS-containing-promoter modules. In addition to TFBSs retrieved from MatInspector, searches in an ~ 1-kb region upstream of the TSS and in the first exon for TFBSs known to be present in *SIRT* promoters of higher vertebrates were performed by ConTra v3 (http://bioit2.irc.ugent.be/contra/v3/#/step/1) [37] using the TRANSFAC database, with sensitivity and accuracy set at core match = 0.95 and matrix match = 0.85. Predictions of CGIs through the entire gene, including a 2-kb region upstream of the TSS, were performed by means of MethPrimer software (www.urogene.org/methprimer/). The search parameters used were length ≥ 200, C + G content ≥50%, ratio of observed/expected CpGs ≥0.60 and window size = 100.

### DNA isolation and bisulfite conversion

Tissue DNA was extracted using the Quick-DNA™ Miniprep Plus Kit (Zymo Research, Irvine, CA, USA) following the manufacturer’s instructions. The quantity and quality of DNA were assessed by a NanoDrop 2000c Spectrophotometer (Thermo Fisher Scientific, Waltham, MA, USA), and DNA integrity was analyzed in a 1% agarose gel. Extracted DNA was bisulfite converted using the EZ DNA Methylation Gold bisulfite conversion kit (Zymo Research, Irvine, CA, USA) following the manufacturer’s instructions.

### PCR of bisulfite-converted DNA

Primers were designed using PyroMark Assay Design 2.0.01.15 (Qiagen, Hilden, Germany) to hybridize CpG-free sites at the highest melting temperature (Additional file 1: Table S1). Reverse primers were labeled with biotin at the 5′-end, and bisulfite-converted DNA was amplified by PCR using Invitrogen™ Platinum™ Taq Hot-Start DNA Polymerase (Thermo Fisher Scientific, Waltham, MA, USA) with forward- and reverse-specific primers at 1 μM each in a total volume of 25 μL. The reaction was performed in a Touchgene Gradient Thermal Cycler (Techne, Cambridge, UK) as follows: 95 °C for 5 min; followed by 35 cycles of 95 °C for 45 s, 60 °C for 45 s, and 72 °C for 1.5 min; with a final extension at 72 °C for 5 min. PCR products were checked by 1% agarose gels to ensure specificity before pyrosequencing.

### Pyrosequencing and DNA methylation analyses

Pyrosequencing analysis was performed as described previously [38]. Briefly, primers for pyrosequencing (Additional file 1: Table S1) were designed using PyroMark assay design 2.0.01.15. The Vacuum Prep Tool (Biotage, Uppsala, Sweden) was used to prepare single-stranded PCR products according to the manufacturer’s instructions. Pyrosequencing reactions were performed in a PyroMark Q24 System version 2.0.6 (Qiagen, Hilden, Germany). Data were analyzed using PyroMark Q24 software, and the quantification of methylation was attained from the average of individual CpGs included in the analyzed sequence.

### Gene expression analyses

RNA was extracted using the MagMAX-96 total RNA isolation kit (Life Technologies, Carlsbad, CA, USA). The RNA yield was 50–100 μg, with absorbance ratios (A260/A280) of 1.9–2.1. RNA integrity number (RIN) values of 8–10 (Agilent 2100 Bioanalyzer; Agilent, Santa Clara, CA, USA) were indicative of clean and intact RNA. Reverse transcription of 500 ng of total RNA was performed with random decamers using a High-Capacity cDNA Archive Kit (Applied Biosystems, Foster City, CA, USA). Negative control reactions were run without reverse transcriptase. A 96-well PCR array of 11 markers of metabolic condition was designed for simultaneous gene expression profiling of liver and WSM. Two housekeeping genes (β-actin and 18S rRNA) and controls for PCR performance were included in each array. Briefly, 660 pg of total cDNA was used in 25 μL PCR reactions. PCR wells contained 2x SYBR Green Master Mix (Bio-Rad, Hercules, CA, USA) and specific primers at a final concentration of 0.9 μM (Additional file 2: Table S2). All pipetting operations for the PCR arrays were performed by an EpMotion 5070 Liquid Handling Robot (Eppendorf, Hamburg, Germany) to improve data reproducibility. Real-time quantitative PCR was carried out in an Eppendorf Mastercycler Ep Realplex (Eppendorf, Hamburg, Germany). The PCR amplification program consisted of an initial denaturation step at 95 °C for 3 min, followed by 40 cycles of denaturation for 15 s at 95 °C and annealing/extension for 60 s at 60 °C. The efficiency of the PCR reactions was consistently higher than 90% and similar among all the genes. The specificity of the reactions was verified by melting curve analysis (ramping rates of 0.5 °C/10 s over a temperature range of 55–95 °C). Negative controls without a template were performed for each primer set. Gene expression was calculated using the delta-delta Ct method [39]. For multigene analysis, all values for a given tissue were referenced to the summer expression level of *sirt1* in + 3 fish, for which a value of 1 was arbitrarily assigned. Fold-changes in gene expression were calculated as the expression ratio between + 3/+ 1 fish. A value > 1 indicates higher expression levels in + 3 fish, and values < 1 indicate lower expression levels in + 3 fish.

This manuscript follows the ZFIN Zebrafish Nomenclature Guidelines for gene and protein names and symbols (https://wiki.zfin.org/display/general/ZFIN+Zebrafish+Nomenclature+Conventions).

### Statistical analysis

Normality and equal variance of data were tested by the Shapiro-Wilk and Levene tests, respectively. Changes in DNA methylation at individual CpG sites were analyzed by Student’s t-test. The effect of age and season on the gene expression of *sirts* and related markers in the liver and WSM was analyzed by Student’s t-test and two-way analysis of variance. The relationship between site-specific DNA methylation and gene expression was assessed by Pearson correlation analysis. The significance level was set to *P* < 0.05 for all tests performed. These analyses were performed using SigmaPlot version 13.0 (Systat Software, San Jose, CA, USA). Supervised multivariate analysis partial least-squares discriminant analysis (PLS-DA) was applied using EZ-INFO® v3.0 (Umetrics, Umeå, Sweden) to depict the contribution of analyzed genes to group discrimination. The quality of the PLS-DA model was evaluated by the parameters R2Y (cum) and Q2 (cum), which indicate the fit and prediction ability, respectively. To discard the possibility of overfitting of the supervised model, a validation test consisting of 500 random permutations was performed using SIMCA-P+ v11.0 (Umetrics, Umeå, Sweden). The relative relevance of genes in the discriminant functions was assessed by the variable importance in projection (VIP) values. A VIP score > 1 was considered to be an adequate threshold to determine discriminant variables in the PLS-DA model [40–42].

## Results

### Sirtuin gene structure and regulatory elements

**Fig. 1:**
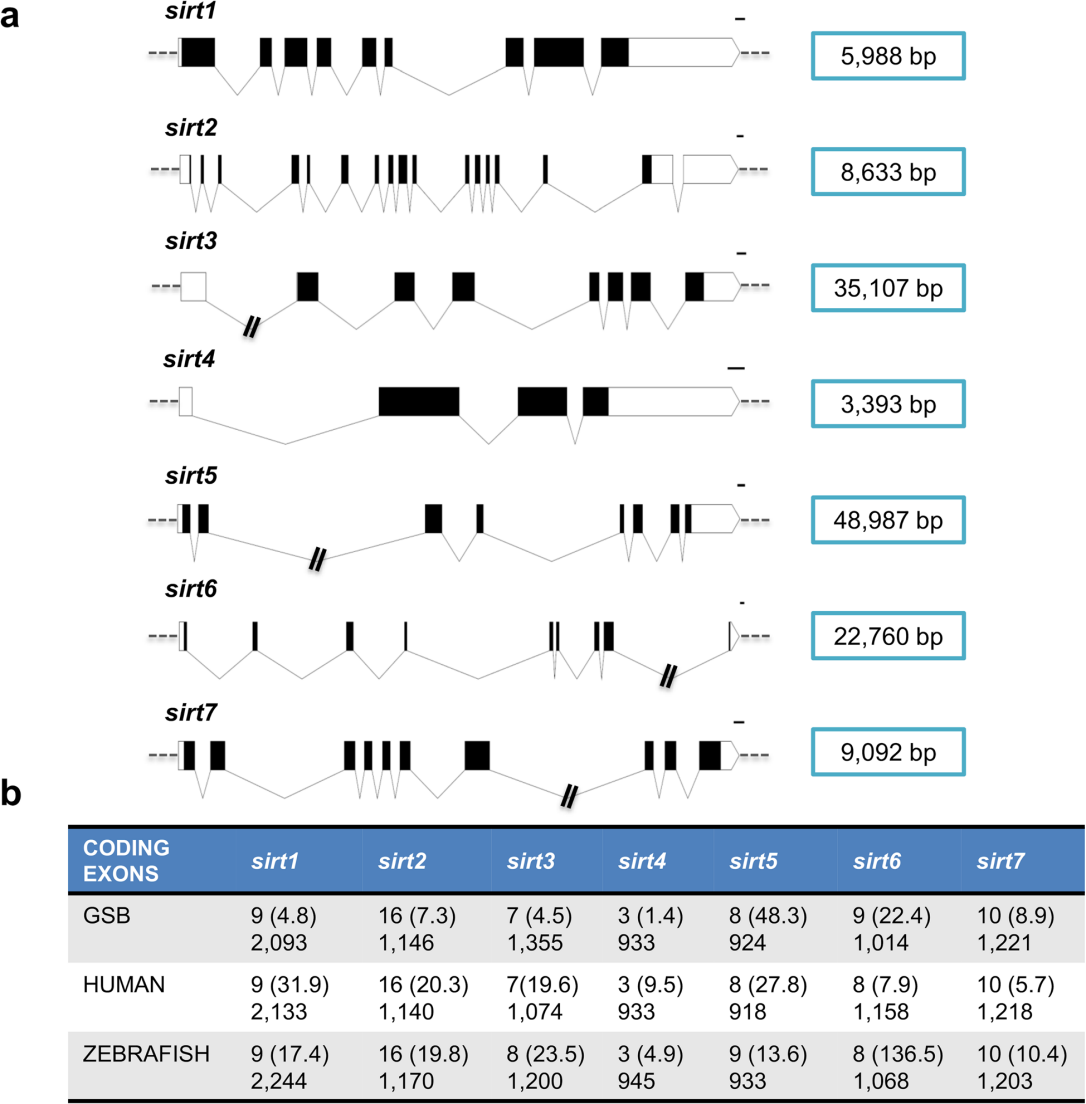
Gene organization of gilthead sea bream *sirts*. **a** Schematic representation of the exon-intron structure of the seven *sirt* genes of gilthead sea bream. White and black boxes represent the noncoding and coding exons, respectively. Introns are shown as connecting lines. Numbers show the total length of the sequences in our database. Scale bars are 100 bp. **b** Number of coding exons and gene size (in kb) from ATG to the stop codon in brackets. Numbers in the second row indicate the total exon size in bp (excluding introns) from ATG to the stop codon. Human and zebrafish sequences were obtained from the Ensembl database (www.ensembl.org)

The seven *sirt* gene sequences of GSB were uploaded to GenBank with accession numbers MN123792-MN123798. These genes have a variable number of exons that range from 3 in *sirt4* to 16 in *sirt2* (Fig. 1a). When comparisons were made within and among the seven *SIRT* orthologous genes of human, zebrafish and GSB, the entire gene length varied from 1.4 kb to 48.3 kb, with the exception of *sirt6* of zebrafish, which contains several long introns that increase the gene length from the start to the stop codon up to 136.5 kb. Despite this, the number and size of exons seems to be highly conserved for each *SIRT* gene through vertebrate evolution (Fig. 1b).

**Fig. 2:**
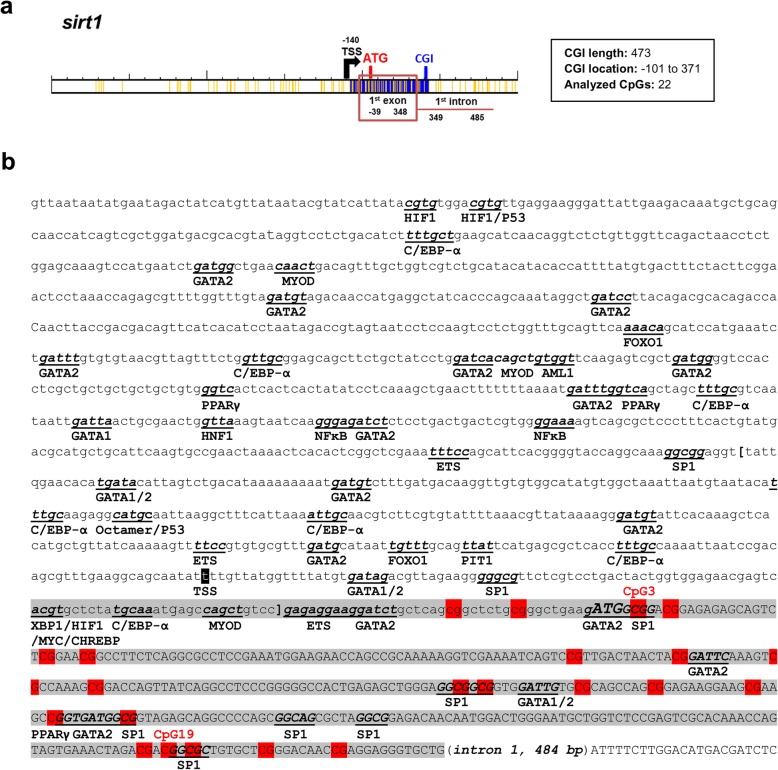
CpG islands and regulatory elements in *sirt1* of gilthead sea bream. **a** Schematic representation of the CpG islands (CGIs) in *sirt1* of gilthead sea bream (in blue). Yellow lines represent CpG sites. Numbers indicate the position of the putative transcription start site (TSS, black arrow) and the starting and ending point of the first exon (red box) and intron (red line) with respect to the start codon (ATG, red). **b** Regulatory elements in *sirt1* of gilthead sea bream. The first intron sequence was replaced by an indication of their length. Lower case letters indicate a 2-kb sequence upstream of the translational start site ATG and include the 5′ UTR and the 5′ flanking region. The predicted core promoter region is flanked by brackets. The putative TSS is shaded in black. Predicted regulatory elements are indicated in bold, underlined, and lower case italics. CGI is shaded in gray. The analyzed CpG positions are shaded in red. This CGI spans the first exon and expands into the first intron (not shown)

**Fig. 3:**
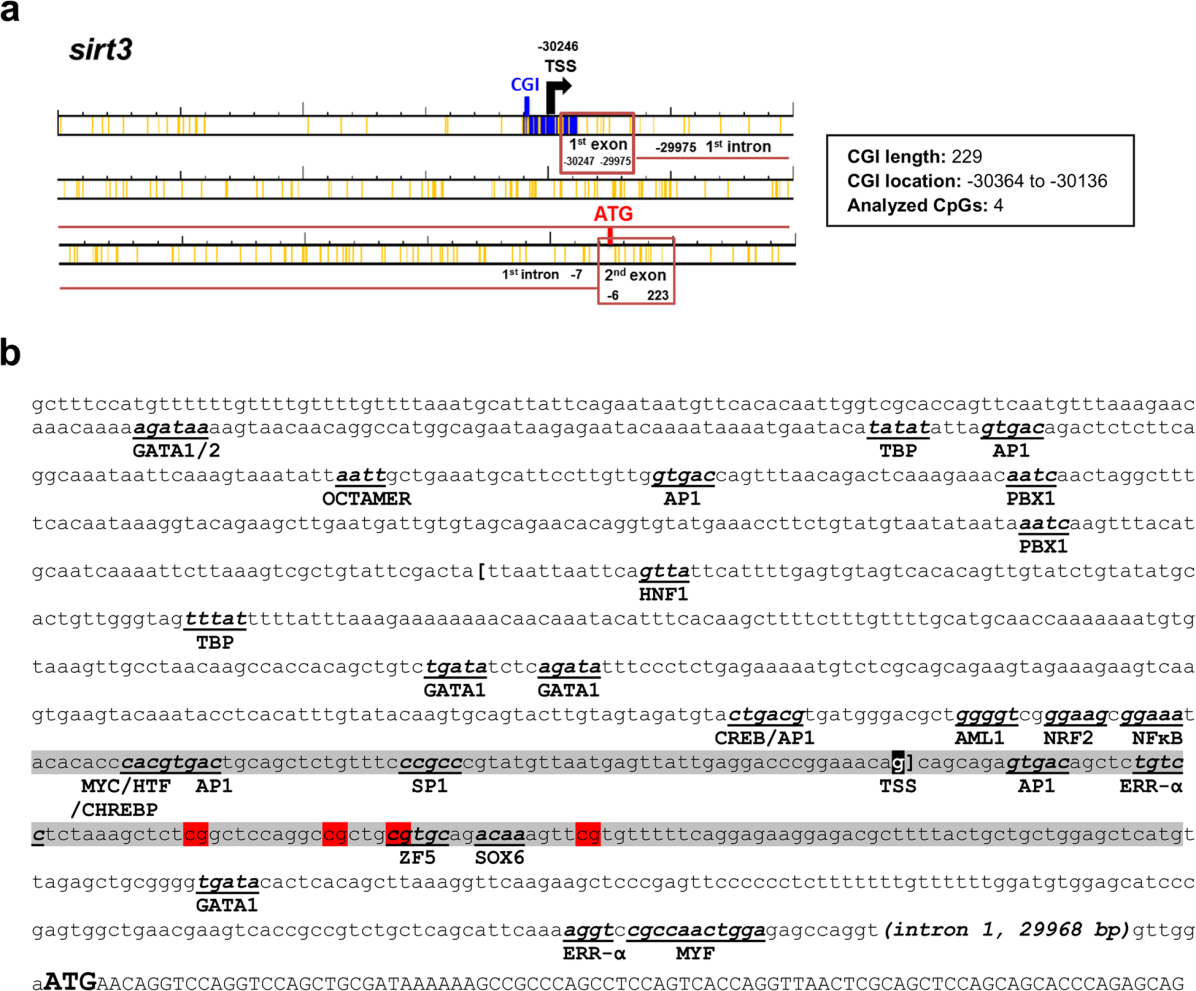
CpG islands and regulatory elements in *sirt3* of gilthead sea bream. **a** Schematic representation of the CpG island (CGI) in *sirt3* of gilthead sea bream (in blue). Yellow lines represent CpG sites. Numbers indicate the position of the putative transcription start site (TSS, black arrow) and the starting and ending point of the first exon (red box) and intron (red line) with respect to the start codon (ATG, red). **b** Regulatory elements in *sirt3* of gilthead sea bream. The first intron sequence was replaced by an indication of their length. Lower case letters indicate a 2.37-kb sequence upstream of the translational start site ATG and include the first exon and the first intron, as well as the 5′ UTR and the 5′ flanking region. The putative TSS is shaded in black. Predicted regulatory elements are indicated in bold, underlined, lower case italics. CGI is shaded in gray. The analyzed CpG positions are shaded in red

The occurrence of CGIs close to TSSs of GSB *sirts* was evidenced in the case of *sirt1* and *sirt3*. The CGI of GSB *sirt1* is 473 base pairs (bp) in length, comprising 100 bp of the 5′ untranslated region (5’UTR), 349 bp downstream of the ATG start codon and 24 bp from the first intron (Fig. 2a). GSB *sirt3* has a shorter CGI (229 bp), which comprises 118 bp from the 5′ flanking region and 111 bp downstream of the TSS, corresponding to the first noncoding exon (Fig. 3a). Further analysis highlighted a *sirt1* gene structure with an open reading frame (ORF) of 2093 bp and a 3’UTR of 1583 bp until the predicted canonical polyadenylation signal. Likewise, *sirt3* contains an ORF of 1355 bp and a 3’UTR of 411 bp until the predicted canonical polyadenylation signal. Searches for regulatory elements in the promoter region of *sirt1* (~ 1 kb upstream of TSS) and in the first exon predict a wide range of multiple *cis*-regulatory elements (i.e., HIF1, P53, C/EBP-α, GATA2, MYOD, FOXO1, AML1, PPARγ, GATA1, HNF1, NF-κB, ETS, SP1, OCTAMER, PIT1, XBP1, MYC and CHREBP) (Fig. 2b). Some of them were also retrieved in the promoter region of *sirt3* (i.e., GATA1, GATA2, OCTAMER, HNF1, AML1, NF-κB, MYC, and SP1), while others seem to be exclusive to *sirt3* (i.e., TBP, AP1, PBX1, CREB, NRF2, HTF, CHREBP, ZF5, SOX6, ERR-α, and MYF) (Fig. 3b).

### Sirtuin 1 and 3 promoter methylation

**Fig. 4:**
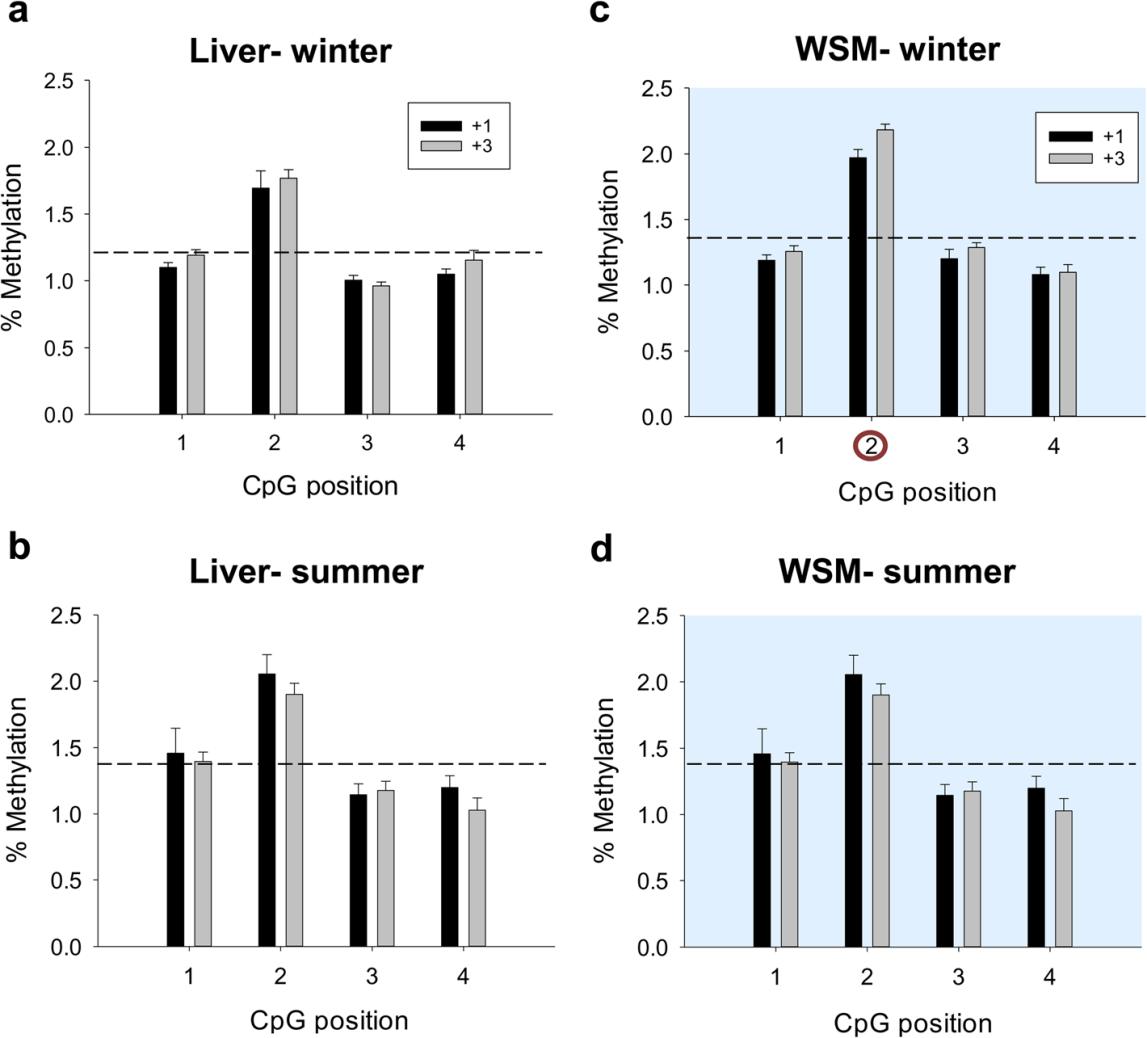
Age and seasonal changes in DNA methylation of gilthead sea bream *sirt3*. Site-specific DNA methylation (%) of *sirt3* in liver (**a**, **b**) and white skeletal muscle (WSM) (**c**, **d**) of fish of different ages (+ 3, three-year-old; + 1, one-year-old) in winter and summer. Data are the mean ± SEM of 8–10 fish. CpG position with a circle indicates significant differences between ages by t-test (*P* < 0.05). Dashed lines indicate the mean methylation of all individuals and positions

**Fig. 5:**
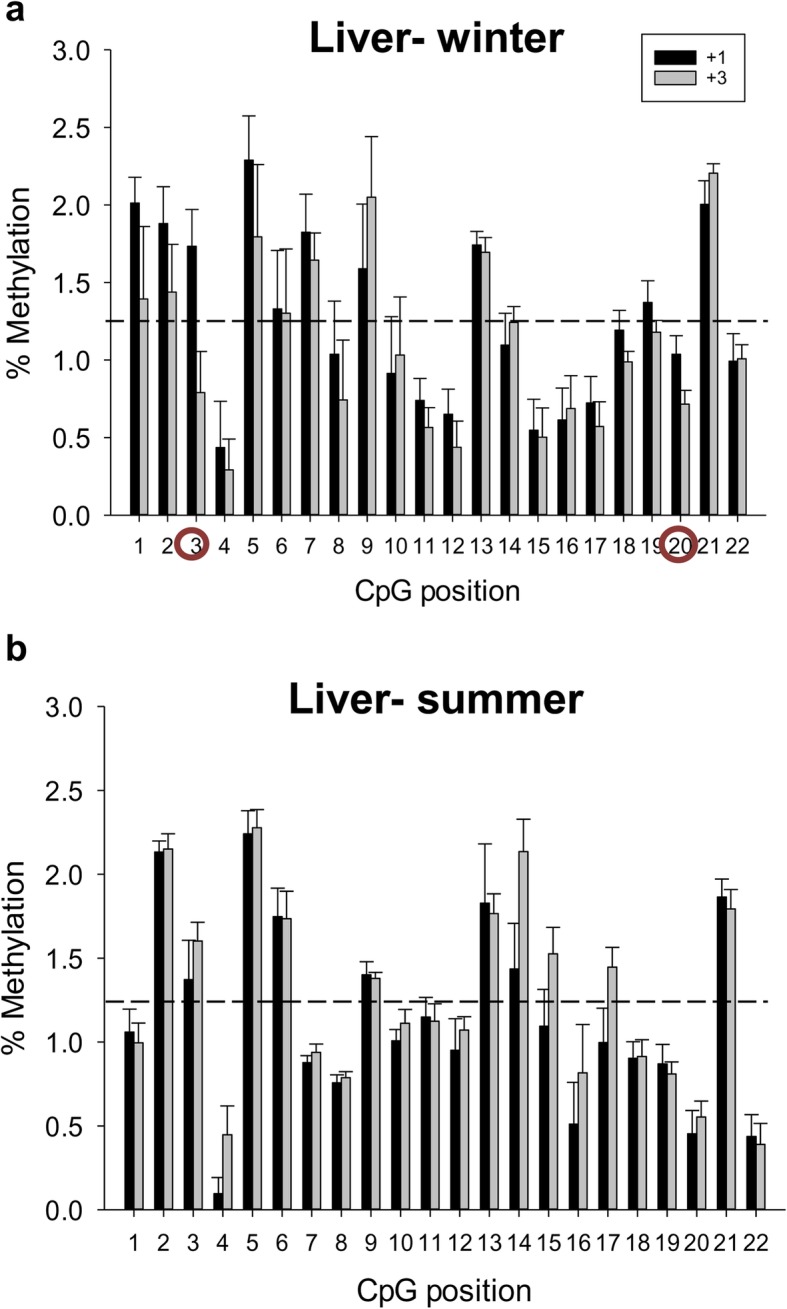
Age and seasonal changes in DNA methylation of *sirt1* in gilthead sea bream liver*.* Site-specific DNA methylation (%) of *sirt1* in the liver in winter (**a**) and summer (**b**) of fish of different ages (+ 3, three-year-old; + 1, one-year-old). Data are the mean ± SEM of 8–10 fish. CpG position with a circle indicates significant differences between ages by t-test (*P* < 0.05). Dashed lines indicate the mean methylation of all individuals and positions

**Fig. 6:**
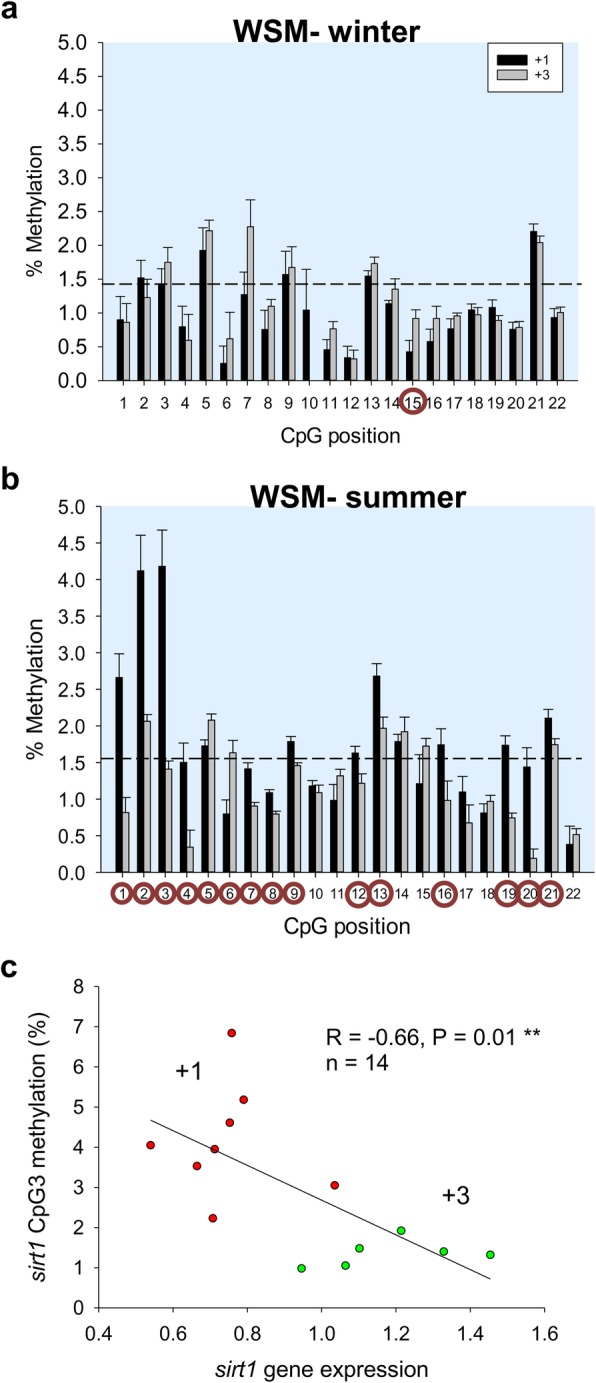
Age and seasonal changes in DNA methylation and *sirt1* expression in gilthead sea bream muscle. Site-specific DNA methylation (%) of *sirt1* in white skeletal muscle in winter (**a**) and summer (**b**) of fish of different ages (+ 3, three-year-old; + 1, one-year-old). Data are the mean ± SEM of 8–10 fish. CpG position with a circle indicates significant differences between ages by t-test (*P* < 0.05). Dashed lines indicate the mean methylation of all individuals and positions. (**c**) Correlation between DNA methylation at CpG3 in the *sirt1* gene promoter region and *sirt1* gene expression in white skeletal muscle (WSM) of gilthead sea bream during summer. Green and red points represent data from + 3, three-year-old and + 1, one-year-old fish, respectively

Up to 4 CpG sites at the CGI of the *sirt3* promoter were chosen for methylation analyses of liver and WSM in fish of two age classes at two critical windows along the production cycle (winter and summer). These CpGs remained generally hypomethylated regardless of age, tissue and season (Fig. 4). However, analyzing individual positions, the CpG2 site consistently showed the highest methylation level, although it did not match with key TFBSs (Fig. 3b). The CGI of the *sirt1* promoter was also hypomethylated without a clear pattern of methylation among the 22 analyzed CpG sites in the liver (Fig. 5) and WSM (Fig. 6). Moreover, this CGI remained hypomethylated when comparisons were made at the hepatic level between the two age groups in both winter and summer (Fig. 5a, b). The same was found in the WSM for fish sampled in winter (Fig. 6a). However, in summer, 15 out of 22 CpG sites (69%) shared a higher methylation in young fish (Fig. 6b). This observation was more evident for the first three CpG sites, with the CpG2 and CpG3 sites reaching the highest level of methylation (~ 4%). The methylation levels of the CpG19 and CpG20 sites were also high in young fish. Of note, SP1 binding sites are close or within all these responsive positions. The involvement of this TFBS in *sirt1* regulation was reinforced by regression and Pearson correlation analyses, which showed across individuals a statistically significant negative correlation between WSM *sirt1* gene expression and the methylation level of the *sirt1* CGI promoter at the CpG3 (*R* = − 0.66; *P* = 0.01) (Fig. 6c) and CpG19 (*R* = − 0.74; *P* = 0.004) sites despite lower methylation levels in the latter.

**Fig. 7:**
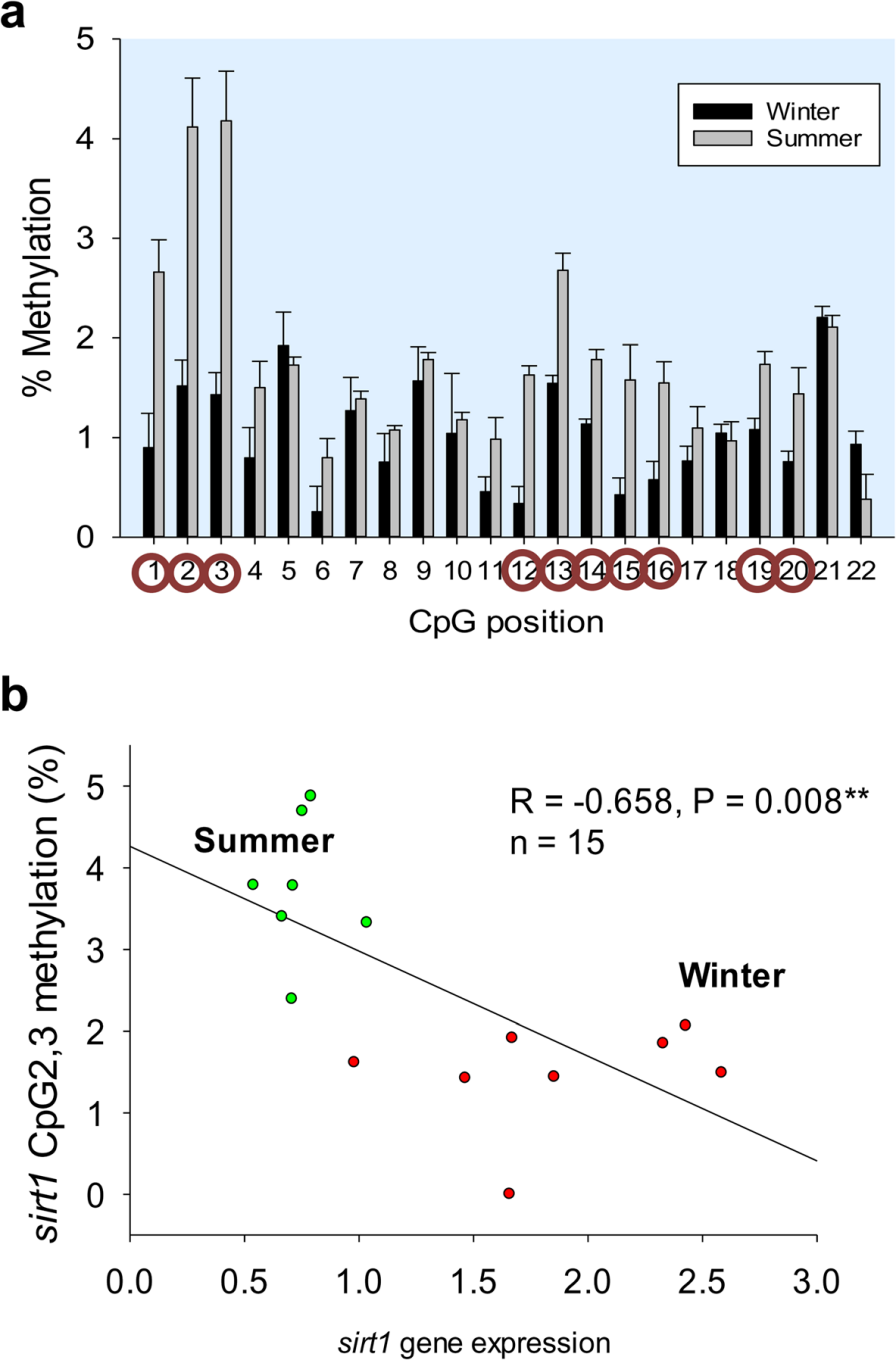
Seasonal local DNA methylation and correlation with *sirt1* gene expression in muscle of one-year-old fish. **a** Site-specific DNA methylation (%) of *sirt1* in white skeletal muscle (WSM) of one-year-old fish in winter (black bars) and summer (gray bars). Data are the mean ± SEM of 8–10 fish. CpG position with a circle indicates significant differences between seasons by t-test (*P* < 0.05). **b** Correlation between mean DNA methylation of SP1-related CpG sites (CpG2, 3) in the *sirt1* gene promoter region and *sirt1* gene expression in white skeletal muscle (WSM) of one-year-old fish. Red and green points represent data from winter and summer, respectively

When comparisons were made on a seasonal basis, the DNA methylation was increased in summer (Fig. 7a). This was especially evident in the + 1 class, and a negative correlation was found between *sirt1* gene expression and the averaged methylation level of the CpG2 and CpG3 sites (*R* = − 0.658; *P* = 0.008) (Fig. 7b). The same trend was reported for the CpG12–14 and CpG16 sites (*R* = − 0.679; *P* = 0.002), with these positions close or within SP1 binding sites. However, changes observed in cytosine methylation at the remaining positions did not correlate with gene expression.

### Gene expression profiling

**Fig. 8:**
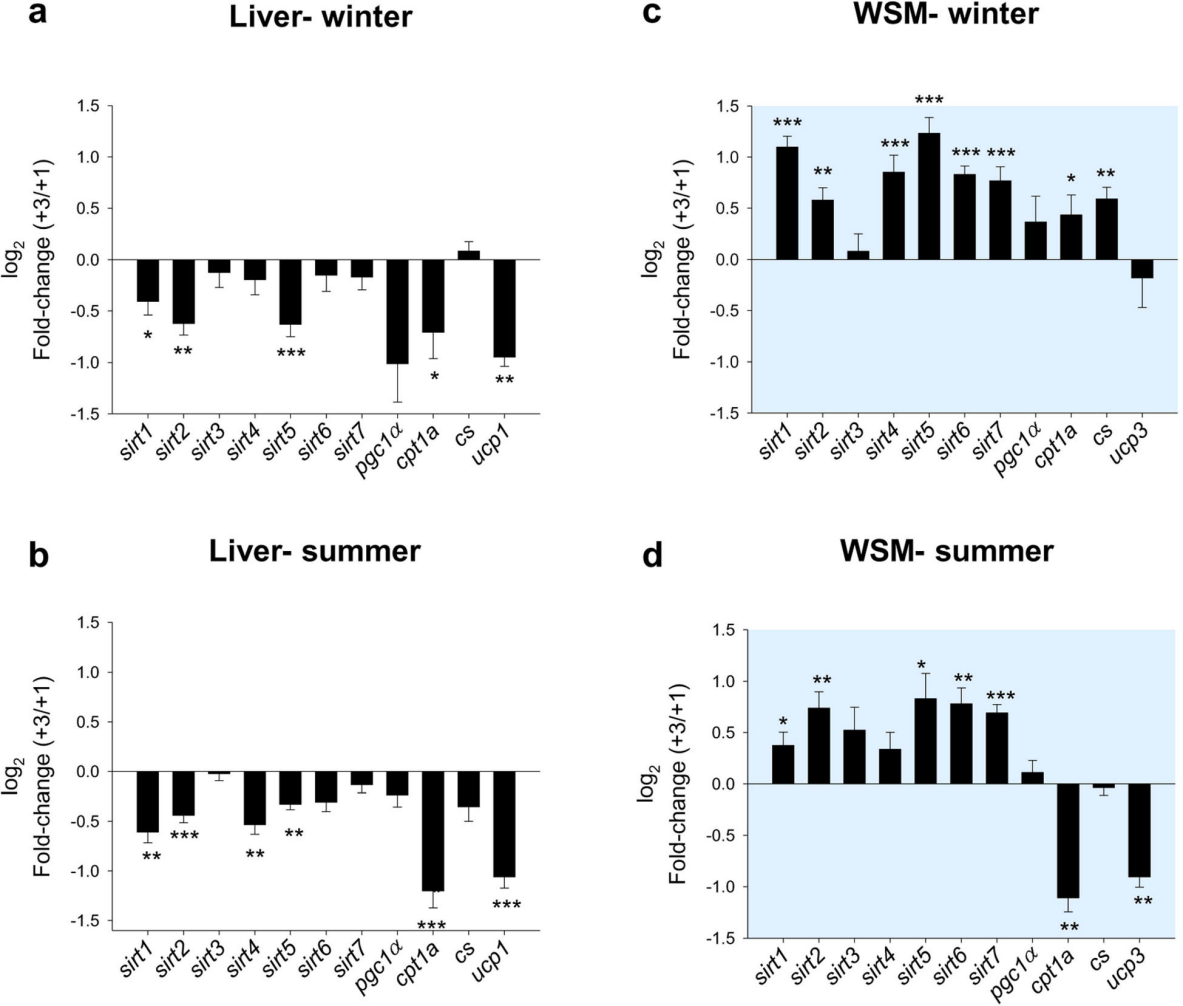
Age and seasonal gene expression changes in *sirts* and related markers in gilthead sea bream. For each season (winter and summer), fold-changes (+ 3, three-year-old/+ 1, one-year-old) in expression in liver (**a**, **b**) and white skeletal muscle (WSM) (**c**, **d**) are shown. Asterisks indicate statistically significant differences (*P < 0.05, **P < 0.01, ***P < 0.001) between ages. Values > 1 indicate upregulated genes in + 3 fish; values < 1 indicate downregulated genes in + 3 fish

Gene expression profiling of all members of the *sirt* gene family in combination with related markers of energy metabolism is summarized in Table 1. A statistically significant effect of season (two-way ANOVA) was found in both liver and WSM for almost all the analyzed genes, with the exception of *sirt7* and *pgc1α* in liver and *sirt2* in WSM. However, the age effects became more evident for *sirt* genes in WSM than in liver. Conversely, markers of fatty acid β-oxidation (*cpt1a*) and mitochondrial uncoupling respiration (*ucp1*) were more responsive to age-mediated changes in the liver than in WSM. Additionally, as a general feature, most genes included in the array were downregulated by age at the hepatic level (Fig. 8a, b). This was especially evident for *sirt1*, *sirt2, sirt5, cpt1a* and *ucp1*, which were consistently downregulated in the + 3 class in both winter and summer. In contrast, in WSM, most of the genes included in the array were upregulated by age in winter (Fig. 8c). This also applied in summer to *sirt1, sirt2, sirt5, sirt6* and *sirt7*, but the opposite was found for *cpt1a* and *ucp3* (Fig. 8d). Of note, most of these age-mediated changes in gene expression were accentuated in winter, as indicated by the statistically significant interaction of age and season in the two-way ANOVA (Table 1).

**Table 1:** Relative gene expression of liver and white skeletal muscle (WSM) of gilthead sea bream. Data are the mean ± SEM of 10 fish of different ages (+ 3, three-year-old; + 1, one-year-old) sampled in winter (W) and summer (S). *P*-values are the result of two-way analysis of variance

	Winter		Summer		Two-way ANOVA		
	W + 3	W + 1	S + 3	S + 1	Season	Age	Interaction
**LIVER**							
*sirt1*	2.53 ± 0.24	3.22 ± 0.21	1.02 ± 0.08	1.52 ± 0.11	**< 0.001**	**0.002**	0.597
*sirt2*	4.15 ± 0.33	6.20 ± 0.46	5.21 ± 0.27	6.99 ± 0.27	**0.012**	**< 0.001**	0.697
*sirt3*	1.54 ± 0.16	1.60 ± 0.12	0.81 ± 0.04	0.82 ± 0.04	**< 0.001**	0.762	0.799
*sirt4*	0.59 ± 0.06	0.64 ± 0.07	0.34 ± 0.02	0.48 ± 0.03	**< 0.001**	0.081	0.406
*sirt5*	6.86 ± 0.54	10.3 ± 0.52	5.47 ± 0.21	6.83 ± 0.26	**< 0.001**	**< 0.001**	**0.014**
*sirt6*	0.69 ± 0.08	0.72 ± 0.05	0.42 ± 0.03	0.51 ± 0.05	**0.001**	0.531	0.935
*sirt7*	1.07 ± 0.08	1.17 ± 0.07	1.11 ± 0.06	1.20 ± 0.04	0.177	0.385	0.795
*pgc1α*	3.29 ± 0.90	5.03 ± 0.63	4.14 ± 0.33	4.75 ± 0.32	0.633	0.058	0.350
*cpt1a*	20.1 ± 3.21	28.7 ± 2.37	3.94 ± 0.48	8.57 ± 0.98	**< 0.001**	**0.004**	0.357
*cs*	30.1 ± 1.95	27.9 ± 1.18	16.3 ± 1.84	19.9 ± 1.32	**< 0.001**	0.666	0.077
*ucp1*	261.4 ± 16.1	496.2 ± 44.8	603.2 ± 45.9	1228.3 ± 105.9	**< 0.001**	**< 0.001**	**0.003**
**WSM**							
*sirt1*	3.90 ± 0.30	1.78 ± 0.17	1.04 ± 0.09	0.77 ± 0.05	**< 0.001**	**< 0.001**	**< 0.001**
*sirt2*	3.23 ± 0.27	2.10 ± 0.14	3.44 ± 0.39	1.96 ± 0.08	0.884	**< 0.001**	0.488
*sirt3*	0.65 ± 0.06	0.58 ± 0.06	0.35 ± 0.06	0.22 ± 0.01	**< 0.001**	0.081	0.548
*sirt4*	0.60 ± 0.06	0.31 ± 0.03	0.39 ± 0.04	0.29 ± 0.03	**0.017**	**< 0.001**	**0.044**
*sirt5*	12.9 ± 1.37	5.19 ± 0.43	5.48 ± 1.02	2.72 ± 0.31	**< 0.001**	**< 0.001**	**0.011**
*sirt6*	0.55 ± 0.03	0.31 ± 0.03	0.32 ± 0.03	0.18 ± 0.02	**< 0.001**	**< 0.001**	0.092
*sirt7*	1.77 ± 0.16	1.00 ± 0.07	0.71 ± 0.04	0.43 ± 0.03	**< 0.001**	**< 0.001**	**0.014**
*pgc1α*	6.32 ± 0.83	4.40 ± 0.80	2.82 ± 0.27	2.55 ± 0.25	**0.002**	0.050	0.500
*cpt1a*	71.6 ± 8.58	49.2 ± 5.78	4.69 ± 0.48	9.78 ± 1.17	**< 0.001**	0.112	**0.020**
*cs*	235.2 ± 19.4	152.0 ± 12.8	63.9 ± 3.11	64.9 ± 4.01	**< 0.001**	**0.002**	**0.006**
*ucp3*	173.0 ± 33.1	165.1 ± 10.8	25.1 ± 1.74	46.1 ± 2.38	**< 0.001**	0.794	0.498

**Fig. 9:**
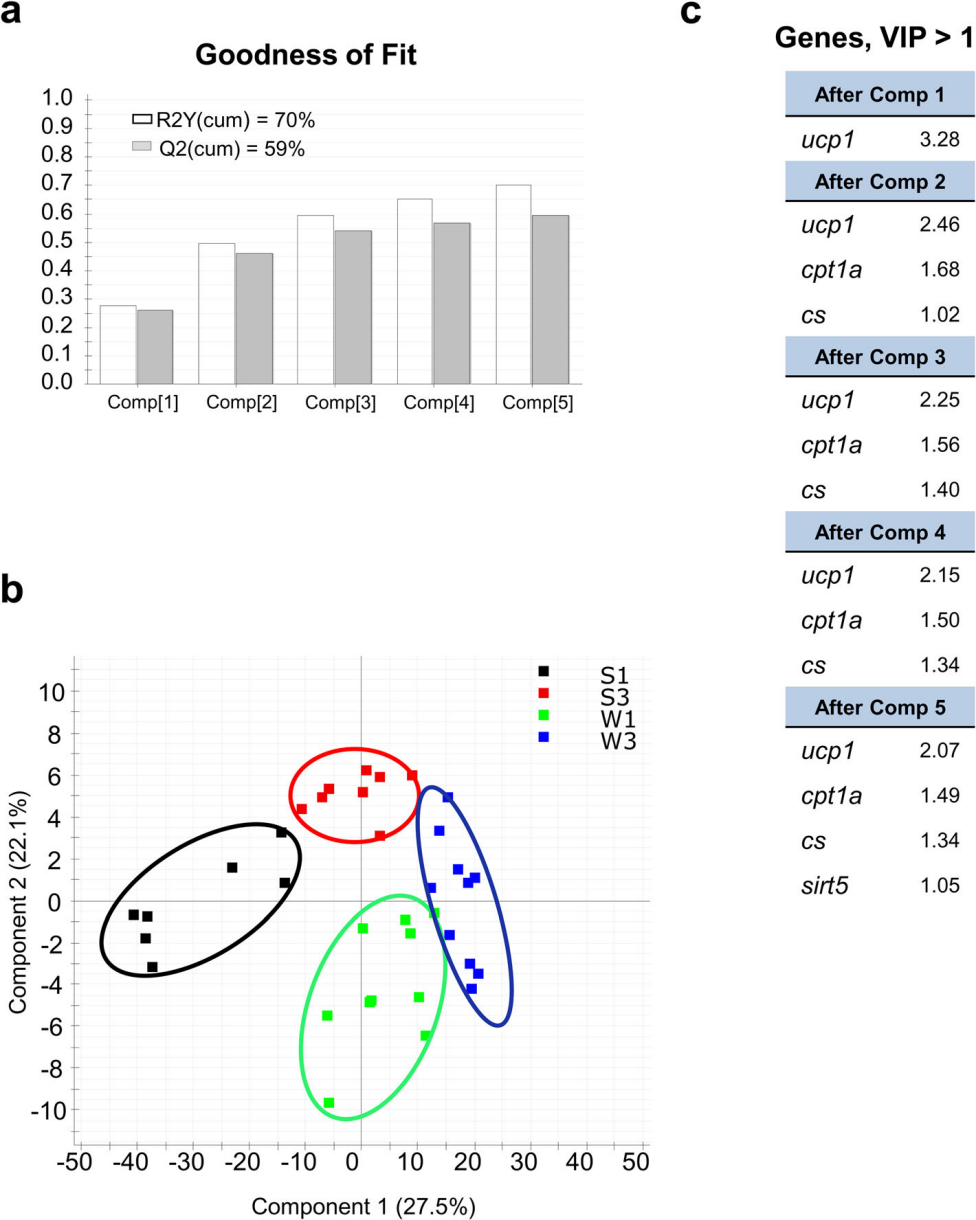
Discriminant analysis (PLS-DA) of molecular signatures in the liver of gilthead sea bream. Data consist of the relative expression of the 11 genes included in the array from fish of different ages (3, three-year-old; 1, one-year-old) in two seasons (summer, S; winter, W). **a** Cumulative coefficients of goodness of fit (R2Y, white bars) and prediction (Q2, gray bars) by each component. **b** PLS-DA score plot of acquired data from fish of + 3 and + 1 years in summer and winter along the two main components, explaining 49.6% of the total variance. **c** Ordered list of markers by variable importance (VIP) in the projection of the PLS-DA model for group differentiation

**Fig. 10:**
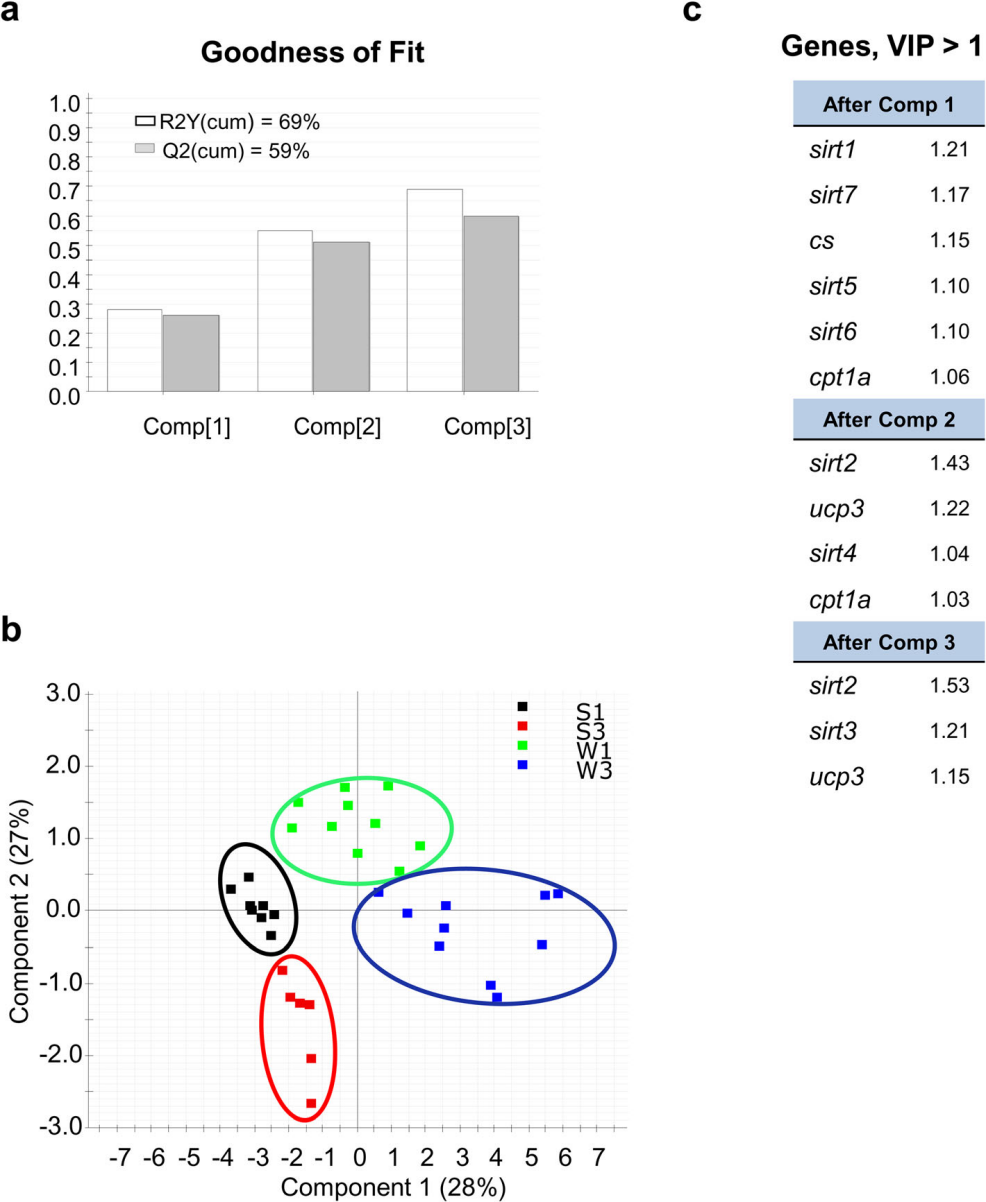
Discriminant analysis (PLS-DA) of molecular signatures in white skeletal muscle of gilthead sea bream. Data consist of the relative expression of the 11 genes included in the array from fish of different ages (3, three-year-old; 1, one-year-old) in two seasons (summer, S; winter, W). **a** Cumulative coefficients of goodness of fit (R2Y, white bars) and prediction (Q2, gray bars) by each component. **b** PLS-DA score plot of acquired data from fish of + 3 and + 1 years in summer and winter along the two main components, explaining 55% of the total variance. **c** Ordered list of markers by variable importance (VIP) in the projection of the PLS-DA model for group differentiation

This temporal and tissue-specific regulation of gene expression is reinforced by multivariate analysis. In liver tissue, *ucp1, cpt1a* and *cs* loadings predicted most of the observed variance, with a poor contribution of *sirts,* which was reduced to *sirt5* after considering five components in the PLS-DA (Fig. 9). In contrast, in WSM, the first three components showed cumulative values for R2Y (explained variance) and Q2 (predicted variance) of 69 and 59%, respectively (Fig. 10a). With this dataset, the separation along the first component explained 28% of the total variance separating groups by season (winter vs summer), whereas component 2 explained 27% of the variance separating groups by age in both winter and summer (Fig. 10b). Genes with a contribution to VIP > 1 in component 1 totaled 6, with a main contribution of *sirt1, sirt5, sirt6* and *sirt7*. When the second component was also considered, a total of 4 genes (*sirt2, sirt4, ucp3* and *cpt1a)* showed VIP values > 1 (Fig. 10c)*.*

## Discussion

### Gene organization of vertebrate *SIRTs* is highly conserved

SIRTs are widely conserved among living organisms with a number that ranges from one in bacteria to seven in vertebrates [43], evidencing this gene expansion is the important role of SIRTs as key components of energy metabolism in all studied living organisms. Certainly, the seven GSB counterparts of mammalian SIRTs have a conserved Rossman fold domain, commonly found in proteins that bind NAD^+^ or NADP^+^ [44]. Furthermore, phylogenetic analysis of GSB Sirts rendered monophyletic clusters [11] according to the four SIRT classes described by [45]. High SIRT conservation across vertebrate evolution is also extensive to gene organization, as illustrated herein by the comparisons made among GSB, zebrafish and human *SIRTs*, which consist of a similar number and size of exons. However, the size of introns for members of the *SIRT* gene family is highly variable within and among species, probably due to the less functional constraints of noncoding DNA regions [46]. Of note, the recognized top TFBSs for human *SIRT1* (C/EBPα, MYOD and MYC) and *SIRT3* (AML1, CREB, HTF, NRF2, PBX1, and TBP) [47] were also retrieved at the predicted promoter regions of GSB *sirt1* and *sirt3*. Furthermore, in different human and rodent metabolic situations, it has been proven that SIRT1 deacetylase activity modulates the function of most transcriptional regulators (e.g., NF-κB, p53, FOXO1, PPARγ, CHREBP, HIF1 and C/EBP-α) forming negative feedback loops [48], which probably also occurs in fish.

### CGIs in *SIRT* promoters appeared early during vertebrate evolution

The number and location of CGIs are very similar in humans and mice [49]. In contrast, the number and density of CGIs are highly variable among fish genomes [50, 51], although interspecies experiments in humans, mice and zebrafish demonstrate that CGIs are an evolutionarily conserved mechanism that protects DNA from methylation to shape the epigenome [52]. This notion fits well with the early appearance of CGIs during vertebrate evolution and is associated with an increased concentration of CGIs close to TSSs from cold-blooded vertebrates to warm-blooded vertebrates [53]. To the best of our knowledge, this general trend has not been assessed in *SIRT* genes, but interestingly, the occurrence of CGIs has been reported in *SIRT* promoters of humans [54–58], mice [59] and ruminants [60–62]. In contrast, in GSB, the occurrence of CGIs was only found in *sirt1* and *sirt3*. With the same search criteria, CGIs were also retrieved in the *sirt1* promoter of fugu (Gene ID: 101061405), zebrafish (Gene ID: 797132), tilapia (Gene ID: 100700447), Atlantic salmon (Gene ID: 106576833) and Australian ghostshark (Gene ID: 103181092), which has a basal position in vertebrate evolution with respect to bony fish. However, the CGI of the GSB *sirt3* promoter did not appear to be conserved in all fish species, which may suggest variations in the regulation of *sirt3* through the fish lineage.

### The presence of CGI- and non-CGI promoters in the GSB *sirt* family

CGIs allow promoter function by destabilizing nucleosomes and attracting proteins that create a transcriptionally permissive chromatin state [49]. Indeed, CGIs colocalize with a great deal of promoters in both the human and mouse genomes [49, 63, 64]. According to this, CGIs should be appropriate for modulating genes that are required to be expressed ubiquitously irrespective of cell type. In contrast, promoters without CGIs should be more suitable for responses to external/internal cues because their transcriptional on/off status could be more strictly controlled depending on the situation [53]. However, this feature is more complex within the *sirt* family in fish, as *sirt* isotypes with/without CGI promoters coexist in GSB, probably to assure ubiquitous and perhaps highly regulated activity in different tissues and metabolic conditions. Certainly, all *sirts* are expressed at detectable levels in 14 analyzed GSB tissues [11], and multivariate analysis highlighted a higher expression level not only of *sirt1* but also of *sirt2* and *sirt5* (without a CGI promoter). However, *sirt1* appeared to be one of the most ubiquitous and highly expressed isotypes when considering as a whole the entire set of analyzed tissues [11]. Conversely, *sirt3,* as well as *sirt4, 6* and *7,* was categorized as a *sirt* isotype with a relatively low expression level, as occurred in a previous study in metabolically active tissues (e.g., liver and WSM) [4], while this *sirt* isotype is able to achieve high expression in immune-relevant fish tissues (e.g., head kidney, posterior intestine) [11, 12]. It is unknown whether CGI promoters may contribute to tissue-specific *sirt3* expression patterns.

### Local DNA methylation might contribute to regulating *sirt1* gene expression

The connection between metabolism and epigenetics through the action of SIRTs has been widely demonstrated in higher vertebrates [9]. In fact, the deacylase activity of SIRTs over histones, TFs and epigenetic enzymes, and their requirement of NAD^+^ as a cosubstrate, makes SIRTs transduce energy metabolic signals into the epigenetic regulation of gene expression, chromatin biology and genome stability [8]. However, the epigenetic regulation of *SIRTs* is less understood, and contradictory results have been reported in different experimental models, with no correlation between *SIRT* expression and local DNA methylation of CGIs in both humans [54–58] and mice [59]. In contrast, a clear negative correlation between gene expression and CGI hypermethylation has been reported for human *SIRT1* [55], and demethylation of bovine *SIRT4–6* promoters enhanced their transcriptional activity, favoring the binding of specific TFs to their promoters [60–62]. In the present study, the analyzed CpG sites of *sirt3* were hypomethylated irrespective of tissue, age and season, which may indicate that the observed changes in *sirt3* expression (mainly related to season) were not regulated in this fish species by changes in DNA methylation at the analyzed CpG sites. At the hepatic level, the 22 examined CpG sites of CGI *sirt1* were also highly refractory to methylation, as occurred in WSM in winter. However, slight changes in DNA methylation were found in the WSM during summer when fish of the + 1 class and the + 3 class were compared, and a negative correlation between *sirt1* expression and DNA methylation was observed at two CpG positions close to SP1 binding sites (CpG3,19). Our results also indicate that the summer decrease in *sirt1* expression is concurrent with the increased DNA methylation at the *sirt1* promoter, which was especially evident in young fish at CpG positions containing SP1 binding sites (CpG2, 3 and CpG12–14, 16).

Despite the above correlations, the magnitudes (fold-changes) of age- and season-related changes observed in *sirt1* gene expression are difficult to be solely explained by the observed changes in a hypomethylated CGI region, which might be indicative of the contribution of other regulatory mechanisms. Indeed, the presence of several TFBSs (e.g., NF-κB, p53, FOXO1, PPARγ, CHREBP, HIF1 and C/EBP-α) at the *sirt1* promoter region, in addition to SP1, may contribute to the regulation of *sirt1* expression independently of SP1-associated methylation. In any case, the association of DNA methylation with an observed phenotype can occur through small differences in the methylation level, often only 1–5% [65]. Indeed a change in methylation of less than 1% affects the binding of NRF1 and E2F1 to the *SIRT6* promoter in bovine adipocytes [62]. Otherwise, it is well known that age-related changes in DNA methylation patterns are characterized in mammals by global genome hypomethylation and region-specific hypermethylation [66, 67]. Although there is currently no proof that changes in specific DNA methylation patterns of *SIRTs* can extend lifespan, it is noteworthy that *SIRT1* is a master regulator of aging as well as inflammation and metabolism [68, 69]. Hence, alterations in the epigenome in adult somatic tissues might reflect aging-associated deleterious events, but developmental changes in the epigenome might be necessary and fine-tuned by environmental cues.

### *sirt* gene expression enables changes in lipogenic and growth energy-demanding processes

The combined gene expression profiling of biomarkers of energy demand (*pgc1α*, *cpt1a*, *cs*, and *ucp1*/*3*) and energy status (*sirt1*–*7*) helps to better discriminate phenotypes with different growth potentials in GSB [4]. Such approaches are also highly informative of the metabolic status of animals across seasons and normal development. In the present study, the hepatic expression of almost all the analyzed markers of energy metabolism was downregulated by age, especially in the cold season. Indeed, differences in feed intake between young and older fish are exacerbated at our latitude from November to March, with voluntary feeding practically stopping in adult fish [70]. Therefore, catabolic states resulting from short-term fasting [11] or natural starvation during winter are sensed by a wide range of energy sensors, including *sirts* that reflect the energy status rather than the energy demand*.* In fact, the most energy-demanding process of the liver is lipogenic activity [71], and reduced lipid biosynthetic capabilities during fasting or temperature drops are linked with pronounced downregulated expression of the enzyme subunits of the mitochondrial respiration chain (oxidative phosphorylation pathway, OXPHOS) [30, 72]. This contrasts with the upregulated expression of markers of OXPHOS in the WSM of fasted or feed-restricted GSB [72, 73], which reflects an increased tissue-energy demand to preserve the protein muscular mass when young fish face a reduced supply of nutrients or an enhanced energy demand for growth during the summer season [70]. This metabolic feature was illustrated herein by increased expression in young fish of markers of fatty acid β-oxidation (*cpt1a*) and muscle respiration uncoupling (*ucp3*), which evolved to protect mitochondria against oxidative stress in a highly oxidative cellular milieu [74, 75]. Certainly, SIRT1 acts as a major repressor of UCP3 in muscle tissues of rodents [76], also inhibiting the progression of different antioxidant responses mediated by NF-κB and NRF2 [48]. Conversely, the downregulation of *SIRT1* enhances the myogenic gene program to adjust it to energetic demands driven by changing growth, nutrient availability or increased muscle activity [77]. Human studies also indicate that SIRT2 enhances myoblast proliferation [78] and differentiation [79], which is related to the enhanced muscle expression of *sirt2* in juvenile GSB with a higher growth potential [4]. Since these findings are apparently contradictory with the observed upregulated expression of *sirt2* with advancing age, we consider that this age-mediated response highlights additional *sirt* functions related in other animal models to genome maintenance and the avoidance of cell senescence [76, 80, 81].

As a corollary of this complex puzzle, multivariate analyses of gene expression patterns indicate that in our experimental setup, changes in *sirt* gene expression at the level of WSM are particularly responsive to physiological changes mediated by age and season. Certainly, six *sirts* out of seven collectively have a discriminant role (in particular *sirt1*) in disclosing the seasonal-related changes in feed intake and growth performance, as well as the switch in metabolism from glucose utilization to fatty acid oxidation, as reported elsewhere [82]. In other words, our study confirms that *sirts* are suited to understanding the adjustment of energy metabolism in GSB, although further studies are needed to fully understand their transcriptional regulation.

## Conclusions

This study demonstrates that the gene structure of *sirts* is highly conserved through vertebrate evolution in the fish lineage. The presence of CGI (*sirt1* and *sirt3*) and non-CGI promoters (*sirt2*, *sirt4*–*7*) was observed in the GSB *sirt* family, and common regulatory elements in the *sirt1* and *sirt3* promoter regions are found in fish and their higher vertebrate counterparts. The gene expression analyses support that GSB *sirts*, especially *sirt1*, are reliable markers of age- and seasonal-related changes in energy metabolism. Correlation analyses of *sirt* gene expression and local DNA methylation were performed for the first time in a marine fish, revealing that a slight increase in local DNA methylation contributes to lower *sirt1* gene expression in WSM. In particular, methylation at CpG positions containing SP1 binding sites might contribute to season- and age-related changes in *sirt1* expression. However, it appears likely that not all changes in *sirt1* gene expression can be explained by DNA methylation at the studied CGI region. This is not surprising given that most changes in energy metabolism require a fast response of fish to cope with a poorly predictable environment, which may involve the participation of several regulatory mechanisms.

## Supplementary information

**Additional file 1 Table S1.** Forward and reverse PCR primers, pyrosequencing primers and sequences for analysis.

**Additional file 2 Table S2.** Forward and reverse primers for liver and white skeletal muscle pathway-focused qPCR array.

## Data Availability

Not applicable.
