# Supplementary material for: Local DNA methylation helps to regulate muscle sirtuin 1 gene expression across seasons and advancing age in gilthead sea bream (Sparus aurata)
Source: Front Zool. 2020 May 15;17:15. doi: 10.1186/s12983-020-00361-1 (PMC7227224; doi:10.1186/s12983-020-00361-1)
Supplement: Supplementary file 1 — Additional file 1 Table S1. Forward and reverse PCR primers, pyrosequencing primers and sequences for analysis. [file 12983_2020_361_MOESM1_ESM.docx]

| **Table S1.**  Forward and reverse PCR primers, pyrosequencing primers and sequence to analyze. | | |  |
| --- | --- | --- | --- |
| **Gene name** | **Primers** | **Sequence** | **CpGs** |
| ***sirt1*** | Forward outer | AGGGATGTATTTATAAAGTTTATATGTTGT |  |
|  | Reverse outer | TTAATAAAACAAACAATTCCCACTC |  |
|  | Forward inner 1p1 (nested) | TGTTTTATGTAAATGAGTTAGTTGT |  |
|  | Reverse inner 1p1 (nested) | CTCCCAACTCTCAATAACCCC *BIOTIN |  |
|  | Pyrosequencing left | GAGAGGAAGGATTTGTTTA |  |
|  | Sequence to analyze left | G**YG**GTTTTG**Y G**GGTTGAAGA TGG**YG**GA**YG**G AGAGAGTAGT TT**YG**GAA**YG**G TTTTTTTAGG | 6 |
|  | Pyrosequencing right | AAAAAGGTRGAAAATTAG |  |
|  | Sequence to analyze right | TT**YG**TTGATT AATTA**YG**GAT TTAAAGT**YG**T TAAAG**YG**GAT TAGTTATTAG GTTTTT | 4 |
|  | Forward inner 1p2 (nested) | GGGTTATTGAGAGTTGGGAGG |  |
|  | Reverse inner 1p2 (nested) | TAATAAAACAAACAATTCCCACTCC *BIOTIN |  |
|  | Pyrosequencing left | TTGAGAGTTGGGAGG |  |
|  | Sequence to analyze left | **YG**G**YG**GTGGA TTGTG**YG**TAG TTAG**YG**GAGA AGGAAG**YG**AA GT**YG**GTGATG G**YG**GTAGAGT AGGTTTTAG | 7 |
|  |  |  |  |
|  | Pyrosequencing right | GTATAAATTAGTAGTGAAATTAGA |  |
|  | Sequence to analyze right | **YG**A**YG**G**YG**TT GTGTT**YG**GGA TAAT**YG**AGGA GGGTGTTGGT ATGTAAAGAG | 5 |
|  |  |  |  |
| ***sirt3*** | Forward outer | ATTGTTGAAATGTATTTTTTGTTGGT |  |
|  | Reverse outer | CCTCACCTACCTAACTCTCCAATTA |  |
|  | Forward inner (nested) | AGAAAGAAGTTAAGTGAAGTATAAATATTT |  |
|  | Reverse inner (nested) | CATAAACTCCAACAACAATAAAAAC *BIOTIN |  |
|  | Pyrosequencing | GTGATAGTTTTGTTTTTTAAAGT |  |
|  | Sequence to analyze | TTT**YG**GTTTT AGGT**YG**TTG**Y G**TGTAGATAA AGTT**YG**TGTT TTTTAGGAGA AGGAGA | 4 |
| *Biotinylated and HPLC purified | |  |  |
