# Supplementary material for: Local DNA methylation helps to regulate muscle sirtuin 1 gene expression across seasons and advancing age in gilthead sea bream (Sparus aurata)
Source: Front Zool. 2020 May 15;17:15. doi: 10.1186/s12983-020-00361-1 (PMC7227224; doi:10.1186/s12983-020-00361-1)
Supplement: Supplementary file 2 — Additional file 2 Table S2. Forward and reverse primers for liver and white skeletal muscle pathway-focused qPCR array. [file 12983_2020_361_MOESM2_ESM.docx]

**Table S2.** Forward and reverse primers for liver and white skeletal muscle pathway-focused qPCR array.

| Gene name | Symbol |  | Primer sequence |
| --- | --- | --- | --- |
|  |  |  |  |
| Sirtuin1 | *sirt1* | F | GGT TCC TAC AGT TTC ATC CAG CAG CAC ATC |
|  |  | R | CCT CAG AAT GGT CCT CGG ATC GGT CTC |
|  |  |  |  |
| Sirtuin2 | *sirt2* | F | GAA CAA TCC GAC GAC AGC AGT GAA G |
|  |  | R | AGG TTA CGC AGG AAG TCC ATC TCT |
|  |  |  |  |
| Sirtuin3 | *sirt3* | F | CTG CCA AGT CCT CAT CCC |
|  |  | R | CTT CAC CAG ACG AGC CAC |
|  |  |  |  |
| Sirtuin4 | *sirt4* | F | GGC TGG CGG AGT CGG ATG |
|  |  | R | TCC TGA ATA CAC CTG TGA CGA AGA C |
|  |  |  |  |
| Sirtuin5 | *sirt5* | F | CAG ACA TCC TAA CCC GAG CAG AG |
|  |  | R | CCA CGA GGC AGA GGT CAC A |
|  |  |  |  |
| Sirtuin6 | *sirt6* | F | ACT CCA CCA CCA CCG ATG TCA A |
|  |  | R | CTC CTC CTC CTT CAC CTT TCG CTT TG |
|  |  |  |  |
| Sirtuin7 | *sirt7* | F | CTG GAG CAA CCT CTA AAC TGG AA |
|  |  | R | CAC CTT CAG ACT GGA GCC TAA |
|  |  |  |  |
| Proliferator-activated receptor gamma coactivator 1 alpha | *pgc1α* | F | CGT GGG ACA GGT GTA ACC AGG ACT C |
|  |  | R | ACC AAC CAA GGC AGC ACA CTC TAA TTC T |
|  | |  |  |
| Citrate synthase | *cs* | F | TCC AGG AGG TGA CGA GCC |
|  |  | R | GTG ACC AGC AGC CAG AAG AG |
|  |  |  |  |
| Uncoupling protein1 | *ucp1* | F | GCA CAC TAC CCA ACA TCA CAA G |
|  |  | R | CGC CGA ACG CAG AAA CAA AG |
|  |  |  |  |
| Uncoupling protein3 | *ucp3* | F | AGG TGC GAC TGG CTG ACG |
|  |  | R | TTC GGC ATA CAA CCT CTC CAA AG |
|  |  |  |  |
| Carnitine palmitoyltransferase 1A | *cpt1a* | F | GTG CCT TCG TTC GTT CCA TGA TC |
|  |  | R | TGA TGC TTA TCT GCT GCC TGT TTG |
|  |  |  |  |
| rRNA 18S | *rRNA 18S* | F | GCA TTT ATC AGA CCC AAA ACC |
|  |  | R | AGT TGA TAG GGC AGA CAT TCG |
|  |  |  |  |
| β-Actin | *actb* | F | TCC TGC GGA ATC CAT GAG A |
|  |  | R | GAC GTC GCA CTT CAT GAT GCT |
